# Accuracy of proton magnetic resonance for diagnosing non-alcoholic steatohepatitis: a meta-analysis

**DOI:** 10.1038/s41598-019-51302-w

**Published:** 2019-10-18

**Authors:** Tae-Hoon Kim, Chang-Won Jeong, Hong Young Jun, ChungSub Lee, SiHyeong Noh, Ji Eon Kim, SeungJin Kim, Kwon-Ha Yoon

**Affiliations:** 1 Medical Convergence Research Center, Wonkwang University, Iksan, 54538 Republic of Korea; 2 Department of Radiology, Wonkwang University School of Medicine, Iksan, 54538 Republic of Korea

**Keywords:** Diagnosis, Magnetic resonance imaging, Medical research, Translational research

## Abstract

Liver biopsy is the reference standard test to differentiate between non-alcoholic steatohepatitis (NASH) and simple steatosis (SS) in non-alcoholic fatty liver disease (NAFLD), but noninvasive diagnostics are warranted. The diagnostic accuracy in NASH using MR imaging modality have not yet been clearly identified. This study was assessed the accuracy of magnetic resonance imaging (MRI) method for diagnosing NASH. Data were extracted from research articles obtained after a literature search from multiple electronic databases. Random-effects meta-analyses were performed to obtain overall effect size of the area under the receiver operating characteristic(ROC) curve, sensitivity, specificity, likelihood ratios(LR), diagnostic odds ratio(DOR) of MRI method in detecting histopathologically-proven SS(or non-NASH) and NASH. Seven studies were analyzed 485 patients, which included 207 SS and 278 NASH. The pooled sensitivity was 87.4% (95% CI, 76.4–95.3) and specificity was 74.3% (95% CI, 62.4–84.6). Pooled positive LR was 2.59 (95% CI, 1.96–3.42) and negative LR was 0.17 (95% CI, 0.07–0.38). DOR was 21.57 (95% CI, 7.27–63.99). The area under the curve of summary ROC was 0.89. Our meta-analysis shows that the MRI-based diagnostic methods are valuable additions in detecting NASH.

## Introduction

Nonalcoholic fatty liver disease (NAFLD) is clinically ‘silent liver disease’ and most patients with NAFLD are asymptomatic until development of cirrhosis and hepatic decompensation^1^. NAFLD is defined as hepatic fat accumulation (≥5% of the liver), ballooning hepatocyte degeneration, inflammatory infiltration of polymorphonuclear leukocytes and progressive fibrosis^2^. NAFLD covers a wide spectrum of disease, including simple steatosis, liver inflammation, fibrosis, and cirrhosis^1,3,4^. A subset non-alcoholic steatohepatitis (NASH) is associated with an increased risk for liver cirrhosis and hepatocellular carcinoma (HCC). Overall, 15% to 25% of patients with NASH progress to cirrhotic liver within 10 years, and 4% to 27% of these patients have HCC^5–7^. A “two-hit theory” explains the progression to NASH. The 1st hit is represented by a fat accumulation in the liver, caused by insulin resistance and obesity-induced sensitization^8^ and the 2nd hit is represented by the progression of hepatic steatosis, which is induced by various sources of inflammation (including proinflammatory cytokines), oxidative stress, and lipid peroxidation^9^. In clinical practice, liver biopsy is the gold standard method for diagnosing NASH. Only NASH patients at high risk (on the basis of increments of aminotransferase level) undergo a liver biopsy. This may lead to under-diagnosis of NASH^10^. Also, the method has well-known weaknesses with the procedure include the invasive approach, sampling errors, inability to assess the severity of cirrhosis, and complications such as pain, bleeding, infection and rarely death^11,12^. Thus, noninvasive diagnostics are warranted in clinical and imaging modalities could have great potentials to distinguish NASH from NAFLD.

Several methods are used for diagnosing NASH. Considerable effort is underway to identify monitoring strategies that noninvasively diagnose NASH using a variety of techniques such as biochemical tests and imaging techniques. Serum biochemistry which assess the metabolic status, provides information about the liver function^13,14^. However, more than two-thirds of NAFLD patients have normal liver function in the studies, and the parameters for liver function are not significantly correlated with histological findings and are not useful for diagnosing NAFLD^15^. Although several patented serum tests can have sensitivity greater than 80%^16,17^, a recent meta-analysis study^18^ derived from eligible 122 literatures reported that no serum biomarkers based on the pooled sensitivity and specificity revealed good (≥80%).

Imaging biomarkers of NASH can noninvasively provide detailed screening information for specific interest or purpose and high diagnostic accuracy suitable for use in large populations. Also, screening large numbers of NASH patients will provide more detailed insight on the mechanisms of pathophysiology and early detection of risk factors in patients. Of the imaging techniques, ultrasound (US) is the first-line imaging test for NAFLD assessment due to its low-cost, wide commercial availability and safety^19,20^. The difference in gray levels on images depends on the acoustic properties (attenuation of acoustic waves, speed of sound, and acoustic impedance) according to different tissue structures. In several studies^21–23^, their sensitivities and specificities for the detection of NAFLD have respectively ranged from 60–94% and 84–95%. And the sensitivities are lower when the degree of NAFLD is mild. Computed tomography (CT) imaging is available because NAFLD decreases the CT attenuation of the liver^24^. A meta-analysis study for detecting the hepatic steatosis reported that the pooled results revealed good sensitivity 81% and specificity 94%. However, these two imaging techniques are not clearly differentiated NASH from simple hepatic steatosis.

Magnetic resonance (MR) technique is a crucial biophysical method for determining the cellular and molecular components^25^. Especially MR imaging (MRI) on the basis of the nuclear MR principles is a robust imaging modality in *in vivo* application to visualize the different image contrasts in normal and abnormal tissues (e.g. simple steatosis, cirrhosis and HCC) for diagnosis^25^. Unlike US and CT, MRI has great merits for screening and staging the NAFLD and/or NASH patients because the method is widely available (as morphology, texture, elastography, strain imaging, diffusion-weighted imaging, perfusion, hepatocellular function and so on) and no ionizing radiation to patients^26,27^. Liver MRI^28^ and MR spectroscopy (MRS)^29^ has been studied for specific mechanism of human NASH pathophysiology, such as fat accumulation, glucose metabolism, inflammation and oxidative stress. A recent meta-analysis study^30^ reported that MRI methods for detecting steatosis or fibrosis in NAFLD had good sensitivity and specificity (≥80%). Taking all of these findings into consideration, MRI and MRS could be considered reliable imaging methods for diagnosing NASH instead of liver biopsy^28,29^. However, the diagnostic accuracy of MRI focusing on NASH in large populations has not yet been evaluated sufficiently. Therefore, this study was conducted a systematic review with meta-analyses to identify the diagnostic accuracy of ^1^H MRI methods for detecting NASH patients with liver biopsy.

## Result

### Study selection and characteristics of included studies

**Figure 1:**
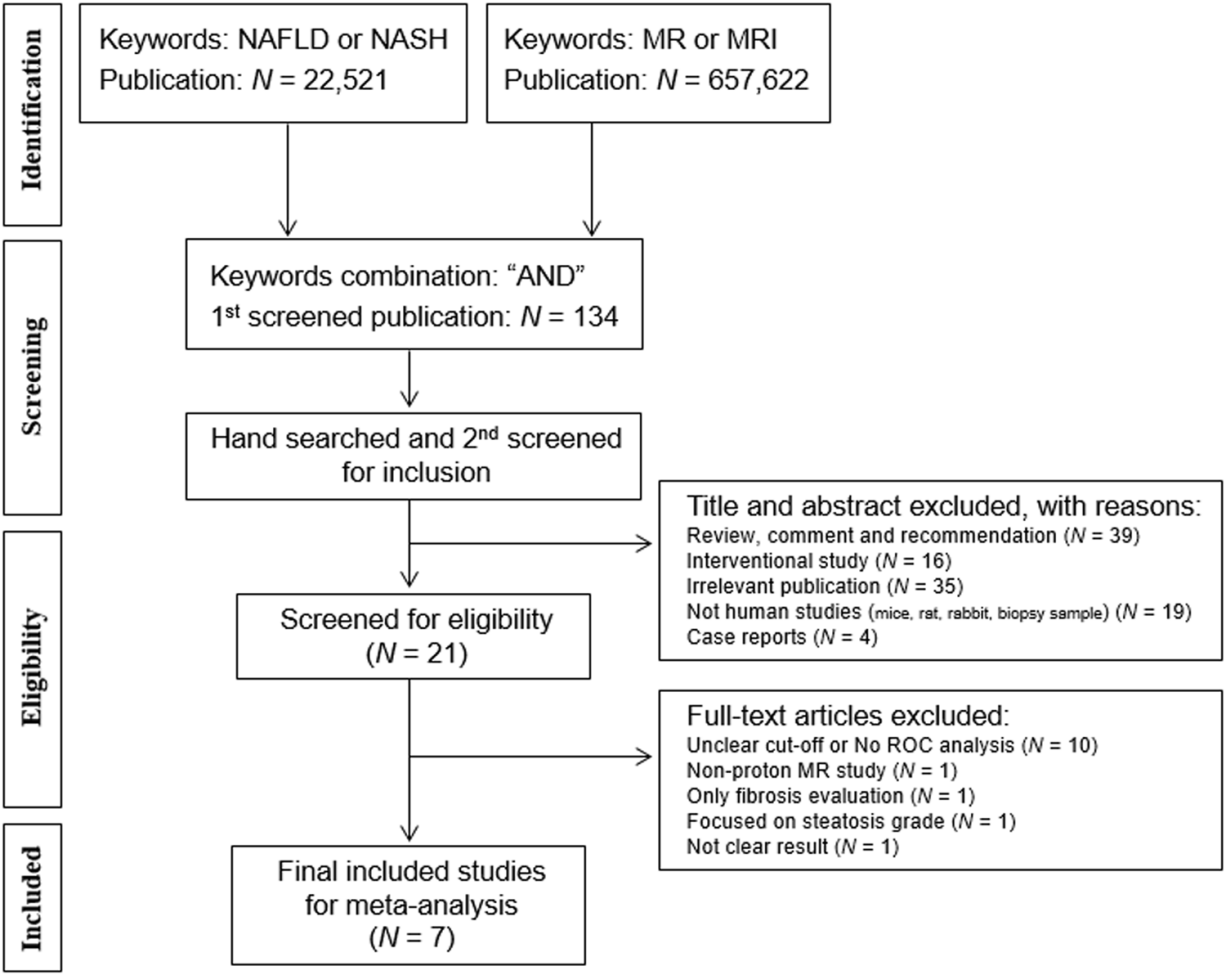
Flowchart showing the process for the inclusion of studies. NAFLD: non-alcoholic fatty liver disease; NASH: non-alcoholic steatohepatitis.

Figure 1 shows flowchart showing the process for the inclusion of studies. Following screening, 134 articles were eligible for title and abstract reviews. Inter-observer agreement was very good (kappa = 0.85). After title and abstract screening, we excluded 113 publications as follows: reviews (n = 39), interventional study (n = 16), irrelevant publications (n = 35), not human studies (n = 19), case reports/series (n = 4). After eligibility screening, 14 articles were excluded with following reasons: no ROC analysis or unclear cut-off (n = 10), only fibrosis evaluation (n = 1), steatosis severity evaluation (n = 1), not clear result (n = 1) and non-proton (phosphorus ^31^P) MR study (n = 1). Finally, 7 studies were included for assessing the diagnostic accuracy of NASH. These studies showed the diagnostic accuracy between biopsy-proven NASH and SS in hepatic fat content (n = 2), liver stiffness (n = 1), contrast enhanced signal intensity (n = 3) and hepatic metabolites (n = 1).

The total number of NAFLD patients in included studies was 485 including of SS 207 and NASH 278. Number of eligible patients analyzed per study ranged from 19 to 190 (median 58). Reported mean age ranged from 38.4 to 55.5 years (mean 48.8 years), while proportion of males ranged from 54% to 79%. Patients were enrolled from the Austria^31^, the United States^32^, Spain^33^, Korea^29^, Netherlands^34^, Japan^35^ and the Australia^36^. The four studies (4/7) used 1.5 T MRI scanners (2 GE, 1 Philips and 1 Siemens) and other studies (3/7) used 3.0 T MRI scanner (2 Philips and 1 Siemens). The characteristics of the included studies are detailed in Table 1.

**Table 1:** Basic characteristics of included literatures.

First author	Year	Location	No. of patients (n)			Age (Mean ± SD)	Gender (M/F)	Scanner	Reference standard	Scoring system
			Both	SS	NASH					
Bastati	2014	Austria	81	46	35	55.5 ± 13	45/36	3.0 T Siemens	Pathology	SAF^d^
Chen	2011	US	58	22	36	51.5 ± ND^a^	ND^a^	1.5 T GE	Pathology	Brunt classification
Gallego-Durán	2016	Spain	87	43	44	50 ± 13	54/33	1.5 T GE & Philips	Pathology	NAS^c^
Kim	2017	Korea	26	15	11	38.4 ± 13	14/12	3.0 T Philips	Pathology	NAS
Smits	2016	Netherlands	24	11	13	54.4 ± 9	17/7	3.0 T Philips	Pathology	NAS
Tomita	2008	Japan	19	9	10	42.0 (38.0–56.5)^b^	15/4	1.5 T GE	Pathology	NAS
Vongsuvanh	2012	Australia	190	61	129	49.5 ± 12	110/80	1.5 T Siemens	Pathology	NAS
			485	207	278					

F: female; M: male; NASH: nonalcoholic steatohepatitis; SD: standard deviation; and SS: Simple steatosis.

^a^ND: not documented; ^b^The value presented median (25th percentile–75th percentile); ^c^NAS indicated the NAFLD activity score; and ^d^SAF system indicated the semiquantitative scoring of steatosis (S), activity (A), and fibrosis (F).

**Figure 2:**
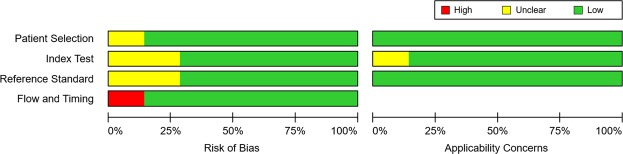
QUADAS-2 assessment findings.

All studies used clinical for the purposes of calculating the test characteristics of MRI to diagnose NASH together with the liver biopsy reference standard. Using the QUADAS-2 assessment tool, all included studies were considered at almost low risk of bias (Fig. 2).

### Diagnostic accuracy for diagnosing NASH

**Figure 3:**
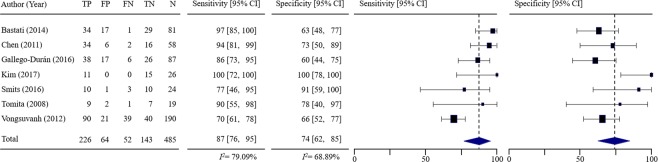
Pooled sensitivity and pooled specificity of included studies. The first column includes the last name of the first author for each of the included studies as well as the year of publication in parenthesis, listed in alphabetical order of author’s last name. The next five columns show the number of true positive (TP), false positive (FP), false negative (FN), true negatives (TN) and total number of patients (N) for each of the studies. Sensitivity and specificity are depicted numerically and then graphically as forest plots. In forest plots, each solid square represents an eligible study. The size of the solid square reflects the sample size of each eligible study. Error bars represents 95% CI.

**Figure 4:**
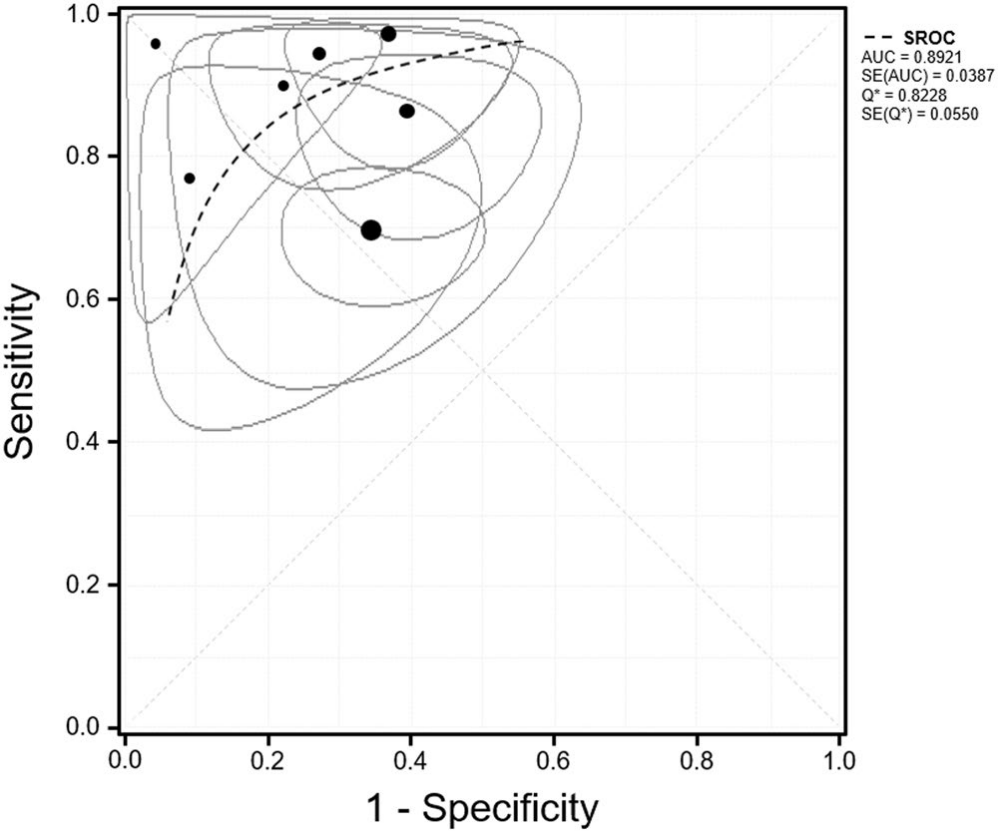
Summary receiver operating characteristics (SROC) curve (dashed line). Black circles depict the sensitivity and specificity of individual studies included in this analysis. The ellipse shows the 95% CI for each study. The size of the circle is proportionate to the number of patients enrolled for each study.

Figure 3 shows forest plots of the sensitivity and specificity of the included studies. Since inter-study heterogeneity existed (I^2^ = 79.09% for sensitivity and I^2^ = 68.89% for specificity), random-effects model was applied to the overall analysis. Sensitivity ranged from 70% to 100% (median 90%) and specificity ranged from 60% to 100% (median 73%). The pooled sensitivity from random-effects regression was 87.4% (95% CI, 76.4–95.3%) and the pooled specificity was 74.3% (95% CI, 62.4–84.6%). Table 2 lists the estimated positive likelihood ratio (LR+), negative LR (LR−) and diagnostic odds ratio (DOR). Pooled LR+ was 2.59 (95% CI, 1.96–3.42) and LR− was 0.17 (95% CI, 0.07–0.38). LR+ and LR− values show weak diagnostic evidence. The DOR was calculated 21.57 (95% CI, 7.27–63.99). Figure 4 shows the summary receiver operating characteristic (SROC) curve for diagnosing NASH in the included studies and its area under the curve (AUC) of SROC curve was 0.8921, indicating very good accuracy.

**Table 2:** Pooled test characteristics.

	All studies (n = 7)
Sensitivity	0.87 (0.76–0.95)
Specificity	0.74 (0.62–0.85)
LR+	2.59 (1.96–3.42)
LR−	0.17 (0.07–0.38)
DOR	21.57 (7.27–63.99)
PPV	0.81
NPV	0.69

The results are reported as point estimates with 95% confidence intervals. LR+ = positive likelihood ratio, LR− = negative likelihood ratio, DOR = diagnostic odds ratio (=LR+/LR−), PPV = positive predictive value, NPV = negative predictive value.

## Discussion

**Figure 5:**
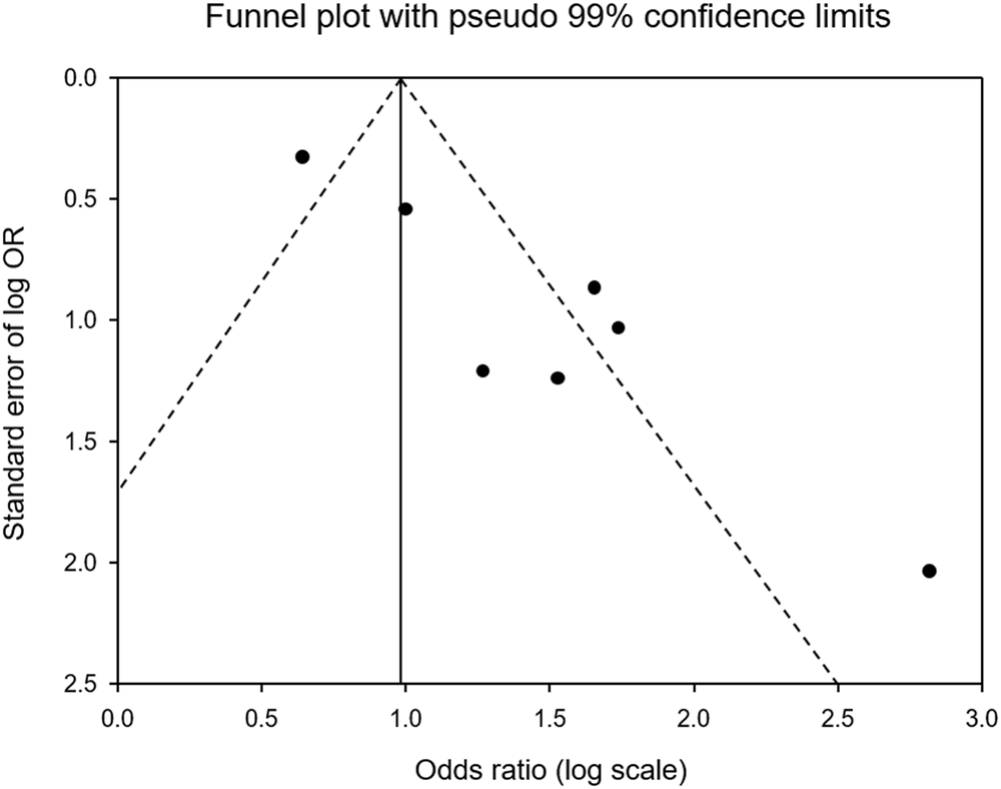
Funnel plot with pseudo 95% confidence limits for assessment of publication bias.

This study was performed a systematic review and meta-analysis of ^1^H MR studies to noninvasively diagnose liver-biopsy proven NASH since 2000. Through the present investigation, 7 studies met eligibility criteria. For the diagnostic accuracy of NASH using MRI, we used the DerSimonian-Laird random-effects model and meta-analytical method for the correlation analysis of sensitivity and specificity. These findings demonstrated that the pooled sensitivity was higher than 80%, but it was significant heterogeneity. Also, publication bias existed for main finding using funnel plot as shown in Fig. 5. The AUC (>0.89) of SROC demonstrated that ^1^H MRI is very good for the diagnostic accuracy of NASH. Although this study is limited for concrete finding by small number of eligible studies, the ^1^H MRI in differential diagnosis of NASH might be considered to use in clinical practice.

In the past decade, there have been efforts for noninvasive diagnosis of NAFLD. Several studies have reported the use of noninvasive imaging techniques including of US, CT, MRI, and MRS as a possible alternative of liver biopsy by evaluating hepatic steatosis^37,38^. These studies have been quantified hepatic fat contents in NAFLD. However, the sensitivities and specificities in these studies vary substantially. Among them, liver MRI has been evaluated for specific mechanism of NASH pathophysiology, such as fat accumulation, glucose metabolism, inflammatory injury and oxidative stress^29,39^. However, an adequately powered, prospective study assessing MRI in terms of reference imaging modality has not yet been performed. Present meta-analysis study can provide the evidence about this gap in knowledge.

Prior to our meta-analysis, several studies have focused on this topic, differential diagnosis of simple steatosis and NASH. Serum biochemical tests for the liver function test are frequently used as indicators of the comprehensive metabolic profile^13,14^. A recent study^18^ reported that no serum biomarkers revealed good sensitivity and specificity. Moreover, these parameters for liver function are not definitely correlated with histological findings, as well as are not useful for the diagnosis of NAFLD in clinical practice^15^. In several US studies^21–23^, the sensitivities and specificities have ranged from 60–94% and 84–95%, but it is only for detecting the hepatic steatosis. And their sensitivities are lower when the degree of steatosis is mild. CT imaging studies^21,24^ reported that the pooled sensitivity and specificity revealed good (>80%) for detecting the hepatic steatosis. But, all of these studies are not clearly differentiated NASH from simple hepatic steatosis. Present study was performed a meta-analysis from seven studies comprising totally 485 biopsy-proven adult patients. Pooled LR+ (2–5) and LR− (<0.2) indicated that there is weak diagnostic evidence in the differentiation of both NASH and SS patients. DOR showed higher diagnostic accuracy of the MRI test as a NASH patient relative to that in a simple steatosis without inflammation.

     With regard to data quality, our eligible studies were judged at low risk. This meta-analysis included an explanation for data extraction, forest plots for pooling test, and the evaluation for the uncertainty around point estimates. The QUADAS-2 domains (consisting of subject selection, index test, reference standard test, and flow & timing) showed low publication bias. Thus, our meta-analysis would be provided useful information in differentiating NASH from simple hepatic steatosis, unlike previous reports of the diagnostic accuracy of laboratory test^18^, US^21–23^ and CT^24^. Heterogeneity among eligible studies may point out the potential bias or variation, therefore it is essential for any meta-analyses that sources of heterogeneity are tested to identify the potential risk factors by interstudy differences in selection of patient (age, NASH severity, gender and so on), the cutoff-point values for differential diagnosis, clinical conditions (type of drug, initial management, drug dosage, treatment duration and etc.) and imaging setting (type of scanners/pulse sequences) or any combinations with the sources. Also, heterogeneity can be caused by defects in the design of study^40^. Moreover, heterogeneity on diagnostic tests might be influenced by the reliability in measurement and/or duration of follow-up^41^. Drawback in report, analysis and interpretation from results occurred frequently in the assessment of the included literatures. To overcome the problem of heterogeneity, we suggest the standardized study protocol/design, prospective, large-scale cohort studies to clarify unmet needs in clinical interests.

Our findings demonstrated that ^1^H MRI could be a rational alternative to liver biopsy for diagnosing the NASH in hospitals. Especially, ^1^H MRI examinations are noticeably useful for screening the early identification of patients at high risk of NASH and reducing the liver biopsy. However, this study included a number of limitations. First, a variety of aspects, as the different populations used (broad ranges of age and diffuse lesion), the diverse number of contents evaluated (hepatic fat, stiffness, contrast-enhanced signal intensity, and metabolites), and the use of diverse cut-off values for the same content, may give rise to a broad range of results in diagnostic accuracy of NASH. Moreover, another concern refers to the heterogeneity of studies, given the potential differences in diverse methods for collection, transportation and storage of biopsy specimen, pre- & post-processing of MR data, and in the uncertainties in the data stability. Information about these issues is still limited. These factors may lead to disturb precise pooling results in the meta-analysis and cause variations in the results for the imaging marker of NASH. Second, the included case-control studies showed a relatively high prevalence of NASH presenting to the NAFLD with severe symptom at high risk when compared to the general population, concerning NASH prevalence. To enroll the patients with a high probability of having NASH through pre-testing, this is likely to use the scheduled liver biopsy in some studies. This may result in overestimation of diagnostic accuracy (spectrum bias) and restrict the external validity of SROC curve generalized to the target population. Also, patient registry using convenient sampling methods was not sufficiently explained to clarify probability in false-negative results. This may give rise to spectrum bias and information bias in meta-analysis. To overcome this issue, further study should enroll a large-scale relevant cohort to reflect the general population. Third, the difference of MRI protocols in the eligible studies was not sufficiently considered for this study. Therefore, the use of a reference standard imaging would useful for directly comparing the performance of test and clearly elucidating the efficacy of MRI. Fourth, although our study only included pathologically-proven NASH/NAFLD patients, it is required to focus more on hepatic inflammation and hepatocellular injury (ballooning). Further study is needed to clarify MRI-based methods for quantifying these more important histologic features (inflammation and ballooning).

In conclusion, this meta-analysis summarizes the evidence about the accuracy of ^1^H MRI examinations for noninvasively diagnosing NASH, with an overall AUC of 0.89. The pooled results reveal good sensitivity of 87.4% and significant diagnostic performance (LR+ 2.59, LR− 0.17 and DOR 21.57). MRI-based diagnostic methods are valuable additions for detecting NASH. The ^1^H MRI examination would be useful for screening of early identification of NASH and reducing of liver biopsy in clinical. However, it is limited for exclusively use in clinical applications. Replication studies and more standardized study designs are required to be an efficient imaging biomarker for NASH diagnosis in clinical practice.

## Materials and Methods

### Literature search

This study was conducted meta-analyses and systematic reviews according to the Cochrane Collaboration guidelines. A systematic search was conducted in Cochrane library, EMBASE and Medline database (January 2000 until December 2017), with the following keywords based on Medical Subject Headings terms: fatty liver, nonalcoholic fatty liver disease (NAFLD), NAFL or nonalcoholic steatohepatitis (NASH), in any combination with MRI examinations. Each search was restricted to English language publications (Fig. 1).

### Selecting study and extracting data

Following deduplication, two authors (T.-H.K., C.-W.J.) independently screened the titles and abstracts of studies. Full-text articles were then reviewed for potential eligibility by the authors. Disagreement between both authors at any viewpoint for analysis was mediated by a 3rd reviewer (K.-H.Y.). These three reviewers extracted in duplicate data from eligible studies using a pre-designed form. Finally, we extracted data to reconstruct 2 × 2 tables for each MRI examination for every individual study.

### Inclusion and exclusion criteria for eligibility

To be eligible for inclusion, MRI studies were required to diagnose NASH with liver biopsy as the reference standard. Studies included the following values for clinical diagnosis of NASH: sensitivity, specificity, diagnostic odds ratio (DOR), area under the receiver-operating characteristic (AUROC) or diagnostic accuracy. We excluded the following studies: animal, case reports or series report, *in vitro* biopsy sample, non-proton nuclei, reviews and irrelevant publication (alcoholic liver disease; other chronic liver diseases or liver diseases with two or more mixed etiologies except NAFLD). Also, studies focusing only on steatosis and/or fibrosis in NAFLD or not differentiating NASH from simple steatosis were excluded.

### Assessment of methodological quality

The quality of included studies was evaluated based on the Quality Assessment of Diagnostic Accuracy Studies (QUADAS-2) criteria^42^. The QUADAS-2 tool consists of four key domains: patient selection; index test; reference standard; and flow and timing. The domains are evaluated in terms of risk of bias and applicability. Each included article independently assessed by two authors using this tool and disagreement between both authors was resolved by a consensus.

### Statistical analysis and data synthesis

In order to evaluate the diagnostic accuracy of MRI in biopsy-proven NASH, this study was used a bivariate random effects model for the meta-analysis of sensitivity and specificity. All analyses were carried out using Meta-DiSc^®^ software (ver. 1.4; Clinical Biostatistics Unit, Ramón y Cajal Hospital, Madrid, Spain) and meta-analysis of diagnostic accuracy (MADA) package on the R program language (R Foundation, Vienna, Austria). To consider the overall effect size for the pooled analyses, we calculated the inverse variance-weighted average adjusted for effect size of each study.

The procedure of meta-analysis accounts for interstudy variability and possible correlation between sensitivity and specificity. Both sensitivity and specificity were estimated to 2 × 2 tables (true positive, false positive, false negative and true negative results) with 95% confidence intervals (CI). We identified the average operating point and computed average sensitivity, specificity, positive LR (LR+), negative LR (LR−) and DOR (DOR = LR+/LR−). To calculate the pooled sensitivity and specificity, we added 0.5 to all zero cells in two-by-two tables when the value of false positive or false negative is zero in each study. LR+ is the best indicator for ruling-in diagnosis. The higher the LR+ the test is more indicative of a disease. LR− is a good indicator for ruling-out the diagnosis^43^. The evidence of diagnostic tests on the basis of LR+ and LR− values is interpreted as conclusive diagnostic evidence (LR+ >10, LR− <0,0.1), strong diagnostic evidence (LR+ >5, LR−, <0.2), weak diagnostic evidence (LR+ 2–5, LR− 0.2–0.5) and negligible evidence (LR+ 1–2, LR− 0.5–1)^43^. Here, the DOR summarized the diagnostic accuracy of an index test as a single value, describing how many times higher the odds are of obtaining a test positive result in a NASH rather than a simple steatosis^44^.

     The random effects model meta-analyses were chosen for two reasons. The 1^st^ reason was the necessity for a pooling effect of the sizes derived from different diagnostic contents & imaging methods and clinical conditions of NASH, and differentiation cut-off value of MRI used in each study. The 2^nd^ reason, the selection of random effects model was due to the statistical heterogeneity of the outcome data. We fitted the DerSimonian and Laird random effects model to depict SROC curves. To assess inter-study inconsistency, we tested by I^2^ index based on Chi^2^ tests, with I^2^ values higher than 50% or *p* < 0.10 respectively representing high heterogeneity^45^. An area under the curve (AUC) of SROC indicates as follows: 0.9–1.0 = excellent; 0.8–0.9 = very good; 0.7–0.8 = good; 0.6–0.7 = sufficient; and 0.5–0.6 = bad^44^. To assess publication bias, inverted funnel plots of the logarithmical OR from each study were plotted against the sample size^46^. All the data are expressed as weighted effect sizes with 95% CI (Supplementary Figures about effect size can be found in supporting information file).

## Supplementary information

Supplementary Information accompanies this paper at https://doi.org/10.1038/s41598-019-51302-w.
