# Supplementary material for: Accuracy of proton magnetic resonance for diagnosing non-alcoholic steatohepatitis: a meta-analysis
Source: Sci Rep. 2019 Oct 18;9:15002. doi: 10.1038/s41598-019-51302-w (PMC6802098; doi:10.1038/s41598-019-51302-w)
Supplement: Supplementary file 1 — Supplementary Information accompanies this paper at https://doi.org/10.1038/s41598-019-51302-w. [file 41598_2019_51302_MOESM1_ESM.docx]

**Supplementary Information**

**Article in *Scientific Reports***

**Accuracy of proton magnetic resonance for diagnosing non-alcoholic steatohepatitis: a meta-analysis**

Tae-Hoon Kim, Chang-Won Jeong, Hong Young Jun, ChungSub Lee, SiHyeong Noh, Ji Eon Kim, SeungJin Kim, and Kwon-Ha Yoon

**Supplementary Figure S1:** A forest chart showing the overall effect size of the Sensitivity obtained by the pooling of effect sizes reported in the individual studies.

| Study | Sample size | Proportion (%) | 95% CI | % Weight |
| --- | --- | --- | --- | --- |
| Bastati, 2014 | 35 | 97.143 | 85.083 to 99.928 | 12.59 |
| Chen, 2011 | 36 | 94.444 | 81.336 to 99.320 | 12.95 |
| Gallego-Duran, 2016 | 44 | 86.364 | 72.649 to 94.827 | 15.83 |
| Kim, 2017 | 11 | 100.000 | 71.509 to 100.000 | 3.96 |
| Smits, 2016 | 13 | 76.923 | 46.187 to 94.962 | 4.68 |
| Tomita, 2008 | 10 | 90.000 | 55.498 to 99.747 | 3.60 |
| Vongsuvanh, 2012 | 129 | 69.767 | 61.064 to 77.539 | 46.40 |
| Total (fixed effects) | 278 | 82.345 | 77.412 to 86.590 | 100.00 |
| Total (random effects) | 278 | 87.409 | 76.469 to 95.279 | 100.00 |

## Test for heterogeneity

| Q | 28.6961 |
| --- | --- |
| DF | 6 |
| Significance level | P = 0.0001 |
| I^2^ (inconsistency) | 79.09 % |
| 95% CI for I^2^ | 57.04 to 89.82 |

**Supplementary Figure S2:** A forest chart showing the overall effect size of the Specificity obtained by the pooling of effect sizes reported in the individual studies.

| Study | Sample size | Proportion (%) | 95% CI | % Weight |
| --- | --- | --- | --- | --- |
| Bastati, 2014 | 46 | 63.043 | 47.548 to 76.793 | 22.22 |
| Chen, 2011 | 22 | 72.727 | 49.778 to 89.271 | 10.63 |
| Gallego-Duran, 2016 | 43 | 60.465 | 44.410 to 75.023 | 20.77 |
| Kim, 2017 | 15 | 100.000 | 78.198 to 100.000 | 7.25 |
| Smits, 2016 | 11 | 90.909 | 58.722 to 99.770 | 5.31 |
| Tomita, 2008 | 9 | 77.778 | 39.991 to 97.186 | 4.35 |
| Vongsuvanh, 2012 | 61 | 65.574 | 52.305 to 77.274 | 29.47 |
| Total (fixed effects) | 207 | 69.885 | 63.259 to 75.951 | 100.00 |
| Total (random effects) | 207 | 74.282 | 62.373 to 84.596 | 100.00 |

## Test for heterogeneity

| Q | 19.2863 |
| --- | --- |
| DF | 6 |
| Significance level | P = 0.0037 |
| I^2^ (inconsistency) | 68.89 % |
| 95% CI for I^2^ | 31.40 to 85.89 |
